# A Model for Tacit Communication in Collaborative Human-UAV Search-and-Rescue

**DOI:** 10.3390/e23081027

**Published:** 2021-08-10

**Authors:** Vijeth Hebbar, Cédric Langbort

**Affiliations:** Coordinated Science Laboratory, University of Illinois Urbana-Champaign, Champaign, IL 61820, USA; langbort@illinois.edu

**Keywords:** signaling, human robot interaction, game theory

## Abstract

Tacit communication can be exploited in human robot interaction (HRI) scenarios to achieve desirable outcomes. This paper models a particular search and rescue (SAR) scenario as a modified asymmetric rendezvous game, where limited signaling capabilities are present between the two players—rescuer and rescuee. We model our situation as a co-operative Stackelberg signaling game, where the rescuer acts as a leader in signaling its intent to the rescuee. We present an efficient game-theoretic approach to obtain the optimal signaling policy to be employed by the rescuer. We then robustify this approach to uncertainties in the rescue topology and deviations in rescuee behavior. The paper thus introduces a game-theoretic framework to model an HRI scenario with implicit communication capacity.

## 1. Introduction

As humans and autonomous systems increasingly interact in a host of physical settings and contexts, there is a growing need for mechanisms and algorithms that facilitate their engagement and understanding. One possible approach to do so, especially in situations where both parties must actively collaborate (as opposed to just co-exist, share space, or merely get out of each other’s way) is by establishing communication, as a way to share information and intent.

Although it is often possible for an autonomous systems to rely on traditional communication devices and protocols (e.g., displaying messages on a screen), many robots also offer the possibility of tacit communication, whereby motions and paths are themselves used as means to encode a desired meaning. This kind of communication amounts to what is known as “signaling” in the economics and decentralized control literature (Grover and Sahai [1], Sobel [2]), since actions are not taken solely because of how they drive the robot to move, but also because of the information contained in that motion. Examples of motion planning specifically accounting for this effect include work by Baillieul and Özcimder [3] and Santos and Egerstedt [4] and, more recently and more broadly, in the growing field of motion legibility (Dragan et al. [5,6]). Indeed, such “legible” motion planning takes into account the inferences an observer makes when viewing the trajectory taken by a robot.

Once one starts exploring the idea of using motion to communicate intent or information, it seems natural to go one step further and consider planning an autonomous system’s motion to specifically and strategically influence its human collaborator’s actions. Keeping with our economics analogy, this amounts to going from “signaling” to “persuasion”, where the latter is concerned with sending messages to actively shape a receiver’s posterior about a state of the world of interest, with the goal of triggering a particular sender- or group-beneficial action (Kamenica and Gentzkow [7]). This kind of problem is typically formulated as a Stackelberg game between sender (in this case, the robot) and receiver (the human agent), with the former acting as a leader, which chooses its messages to influence the receiver’s actions. In this paper, we consider such a Stackelberg tacit communication design problem in the context of search-and-rescue missions.

Unmanned Aerial Vehicle (UAV) usage in search and rescue (SAR) applications has been extensively studied in recent years. The primary challenge that is addressed by these UAVs is to quickly sweep large swaths of area with the goal of finding the rescuees and, in certain situations, providing relief in the form of air-drops. Traditionally, such SAR scenarios have been considered in either the ‘full communication setup’, where the rescuee and rescuer/UAV can agree on a meeting location and actively coordinate to rendezvous there, or the more common, ‘no communication setup’, where they are oblivious to each other’s actions (and locations) and try and meet (with high probability) in minimum time. The latter case is akin to a “hide-and-seek” game of one player finding another in a known environment in the minimum time possible. Alpern and Gal [8] discuss and study various strategies for such co-operative rendezvous games between non-communicating players. Keeping in line with the initial discussion we had in the previous paragraph we are more interested in the intermediate (and, arguably, more realistic) scenario where the rescuer/UAV has some limited ability to communicate with the rescuee. The rescuer can transmit some information about its intentions or influence the rescuee to move in a mutually beneficial way.

Recent work has shown that autonomous agents, in particular UAVs, have implicit signaling capabilities allowing them to convey intent in the absence of more formal communication channels. Szafir et al. [9] use modified flying trajectories to signal intent while in another work (Szafir et al. [10]) they show that lights on a quadrotor can be used to convey directionality. Such signaling capabilities are closely tied to the idea of legible motion. Its primary objective is to make the robot’s intent predictable to the human and, thus, increase human comfort in HRI scenarios. In other words, legible motion is the answer to the question: How can a robot use implicit signaling abilities to convey information about its own subsequent motion? However, as indicated earlier in this section, we are interested in taking a step further and answering a naturally arising question: How can a robot perform persuasion, i.e., use such implicit signaling abilities to convey information to a human with a goal of influencing her subsequent behavior?

### 1.1. Motivating Example

**Figure 1:**
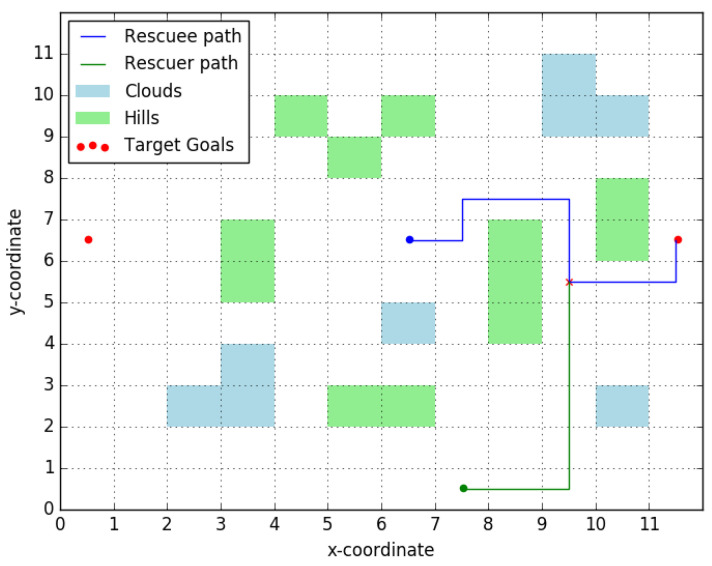
Rescue area topology. Rescuee takes optimal path (blue) to the indicated target. The rescuer picks an optimal rendezvous point (red cross) to meet the rescuee. Clouds (blue shading) act as obstacles to the UAV and hills (green shading) act as obstacles to the rescuee.

To motivate our ideas further we will consider a simple example, one that will be revisited throughout the paper to illustrate key elements and ideas in our work. Consider the simple terrain represented in Figure 1, with two plains (located at the red circles) to the east and west of the rescuee’s initial location (blue circle). The rescuee’s initial location is assumed inaccessible to the rescuer/UAV, initially located at the position in the south marked by a green circle.

If the existence and (approximate) location of the plains is known to both the rescuee and the rescuer, and there are means for the latter to produce a boolean signal, an option to introduce coordination between the two is for the UAV to issue a message signifying its intentions. Consider a message that may be interpreted as, “I am going to the eastern plain”, as a way to indicate the rescuer’s intention to head in that direction. Assuming that the signal motivates the rescuee to move towards the ‘eastern plain’, such a signal holds value even if the rescuer does not actually plan on meeting the rescuee at the plain (but, instead, picks some other accessible point along the path taken by the rescuee to rendezvous). This strategic effect of the transmitted signal is precisely what we wish to capture in our modeling. Note that such signaling is only feasible if the number of salient “landmarks” (here, the plains), each of which corresponds to a message, is small enough for the rescuee to reliably interpret the different messages and for the UAV to be able to produce them.

As evidenced by works of Szafir et al. [9,10] and Dragan et al. [5,6] there exist intrinsic signaling abilities in UAVs. In our work, we merely assume that such capabilities exists (along with the ability to produce a message set of appropriate cardinality) but do not concern ourselves with its physical implementation or specific characteristics, i.e., with *how* the message manifests physically. In the context of our example from Figure 1, this assumption means that there exist two messages which are interpreted by the rescuee as the rescuer’s intent to go to either of the two ‘target’ plains. With this as the background, our aim is to derive efficient algorithms to determine *which* message the rescuer should send to minimize its and the rescuee’s effort.

Our problem is naturally formulated as a Stackleberg game between the rescuer (acting as the leader) and the rescuee (acting as the follower), as a way to account for the fact that each message prompts the rescuee to move towards the corresponding landmark. The notion of legibility based signaling fits consistently in the asymmetric structure of the rescuer–rescuee interaction we are considering in which the rescuer’s message initiates the moves.

To give our problem additional structure and to make it amenable to further analysis we make some preliminary assumptions. Both the rescuer and the rescuee are assumed to have constant velocities over the terrain. In moving across obstacles (such as hills and clouds) the players incur an increased path cost and, thus, take more time to traverse. The constant velocity assumption allows us to work with the path cost and the travel time interchangeably. The rescuee will seek to reach the target in minimum time, or equivalently, minimize its path cost to the goal. The rescuer will try to minimize both the path cost for the rescuee and its own path cost to the point of rendezvous.

In other words, given a signal, the rescuee interprets the goal that the UAV is flying to and plans the shortest path to that goal. The rescuer, modeled as a rational player, assumes the human is going to behave as expected (i.e., takes the shortest path to the perceived goal) and plans out its path to intercept the human’s path. There are multiple implicit assumptions we have made in the game as presented above.

**Assumption** **1.** *The ability of the rescuee to compute the shortest path relies on her knowledge of the precise terrain in the region under consideration. We make this assumption on her knowledge of the topology around her.*

**Assumption** **2.** *The formulation presented above also relies on the rescuee’s ability to precisely compute these shortest paths given the region topology. We assume that the rescuee possesses such abilities.*

**Assumption** **3.** *We assume that the topology surrounding the rescuee is common knowledge.*

**Assumption** **4.** *Finally, the rescuer is assumed to have complete knowledge of the velocities of both the human and itself.*

### 1.2. Roadmap Ahead

Having established our framework, we are specifically interested in answering the following questions

1. How can we arrive at the signaling policy that the rescuer should employ to minimize its cost?;
2. How sensitive is our approach of finding the optimal signaling policy to changes in the velocities of the players? Such situations mean that our Assumption 4 is violated;
3. How sensitive is our approach to uncertainties in the rescuer’s knowledge of the environment topology? This translates to relaxing our Assumption 3;
4. How sensitive is our approach of finding the optimal signaling policy to changes in the rescuee’s path planning model? Such a situation means our Assumption 2 is contradicted;
5. How do we account for such uncertainties in designing a robust approach to find the optimal signaling policy?

In Section 2, we formalize our problem statement and arrive at a signaling approach to be employed by the rescuer when Assumptions 1–4 hold. In other words, this section seeks to answer the first question posed above.

In Section 3, we analyze the sensitivity of the approach developed in Section 2 to uncertainties in the rescuer’s knowledge of the velocities of agents and travel costs over the terrain topology. This part of the work effectively answers the second and third question posed above. In this section, we also look to answer the fourth question by looking at some alternative models of human path planning.

Jan et al. [11] were among the first to empirically show that humans often deviated significantly from shortest paths. Zhu and Levinson [12] went a step further and showed that although humans deviate from shortest path very often, travel time on the path picked by the human is not too far from the least travel time over the network. This suggests that ϵ-shortest path planning, i.e., picking a path which has at most (1+ϵ) times the path length of the shortest path, is a viable alternative model for human motion planning. The study observed that 80% of all non-commute and 70% of commute trips had a travel time that exceeded the least travel time by no more than 20%. Zhu and Levinson [12] also provide a more exhaustive literature review of human path-planning models.

Motivated by evidence for vector based navigation in animals, Bongiorno et al. [13] proposes an alternate theory of line-of-sight based human route planning. As the name suggests, under such a model the human seeks to stay close to the line-of-sight path between her origin and the destination. It is worth noting that all the studies (Jan et al. [11], Zhu and Levinson [12], Bongiorno et al. [13]) presented above look at human route planning in urban environments. An individual may choose to deviate from the optimal path for many reasons in such an environment. For instance, a individual might take a detour to avoid a toll or a particular traffic signal. In a rescue scenario such as ours, it is only more likely that the human chooses the least travel time/shortest path.

In Section 3.3, we will analyze the performance of the approach developed in Section 2 when rescuer plans her path using either of the alternate models presented above.

In Section 4 and Section 5, we seek to answer the final question posed above. In Section 4 we develop a robust counterpart to the signaling policy obtained in Section 2 that accounts for bounded uncertainties in both the travel costs over the graph and the agent velocities. In developing the robust approach, we also present a novel and efficient algorithm to find feasible points for the rescuer to rendezvous with the rescuee, despite the uncertainty in knowing the rescuee’s path.

In Section 5, we will seek to reduce the conservativeness of the fully robust approach developed in Section 4 by exploiting some distributional information we may have about travel-costs over the rescue environment. We will also present an additional modification to our approach in this section that accounts for alternate models of human path planning.

## 2. Optimal Signaling Policy

In line with the illustrative example provided in Section 1 we will consider a discretized terrain (e.g., grid) for the rendezvous problem in this work. Equivalently, we can study the problem as being defined over a directed finite graph G=(V,E). The cost of traversing the edge between nodes *i* and *j* for the rescuee and the rescuer are defined as edge weights $w_{ij}^{r}$ and $w_{ij}^{R}$, respectively. Nodes $v_{r}$ and $v_{R}$ denote the initial position of the rescuee and rescuer, respectively. For the purpose of this section, we assume that the edge-weights, the initial positions of the players and the locations of the target goals are all common knowledge.

Let P denote the set of all paths on the graph. $P_{i\to j}$ denotes the set of all paths starting from node *i* and terminating at node *j*. $\phi_{r}$ and $\phi_{R}$ are real-valued functions defined on P that give the path cost for any path, for the rescuee and rescuer, respectively.

### 2.1. Rescuee Policy

The rescuer, acting as the leader in the Stackelberg game, sends out a message m∈M to the rescuee, indicating the goal node $v_{m}\in V$. The set M is assumed to have much lower cardinality than V. The rescuee then acting as the follower, observes this message and seeks to minimize

(1) $$U_{r}\left(m,P\right)=\phi_{r}\left(P\right)$$

over paths $P\in P_{v_{r}\to v_{m}}$. In other words, the rescuee chooses a shortest path connecting its starting position to the landmark $v_{m}$. Dantzig [14] gives a natural linear program formulation for the shortest path problem. Minimizing (1) is equivalent to solving the linear program,

(2) $$\min_{x_{ij}\geq 0}\sum_{ij\in E}w_{ij}^{r}x_{ij}$$

(3) $$S.T.\;\forall i\;\sum_{j}x_{ij}-\sum_{j}x_{ji}=\left\{\begin{array}{cc} 1 & i=v_{r}\\ -1 & i=v_{m}\\ 0 & \mathrm{otherwise}. \end{array}\right.$$

$x_{ij}$ here can be intuitively seen as an indicator variable for whether the directed edge (ij) is a part of the shortest path. The constraints in (3) are node-wise constraints and balance the inflow and outflow at every node. At the source node ($v_{r}$) the net outflow is 1, indicating that there is no edge of the shortest path going into the source. Similarly, the −1 at terminal node ($v_{m}$) indicates that no edge in the shortest path exits this node.

When the edge weights are known with certainty the linear program in (2) can be solved using the simplex method. The same problem may also be solved using the Dijkstra’s algorithm. The latter approach with its polynomial time complexity is preferred for computation of the shortest path. However, the LP formulation is presented here as it lends itself more conveniently to analysis and we will revisit it in Section 4. Note that the minimizer to (2) need not be unique. In general, the solution to the shortest path problem between two nodes is a directed sub-graph with every path in the sub-graph between those two nodes being a shortest path in the original graph (Hebbar [15]).

**Definition** **1.** *Let $G_{m}=\left(V_{m},E_{m}\right)$ denote the directed sub-graph obtained as the minimizer to (2) and let $P_{v_{r}\to v_{m}}^{m}$ be the set of path between $v_{r}$ and $v_{m}$ in $G_{m}$. We can define the* candidate rendezvous points set *$X_{m}$ as*

$$X_{m}=\left\{v\in V_{m}|v\in P\;\forall P\in P_{v_{r}\to v_{m}}^{m}\right\}.$$

By definition, $v_{m},v_{r}\in X_{m}$ and $X_{m}$ is finite as it is a subset of a finite set V. We distinguish $v_{m}$ as the *terminal rendezvous point*. Note that the rescuee is guaranteed to visit each node in $X_{m}$ regardless of the path she actually uses to reach $v_{m}$. As a result, $X_{m}$ can be considered by the rescuer as the set it is creating for itself by sending message *m*.

**Figure 2:**
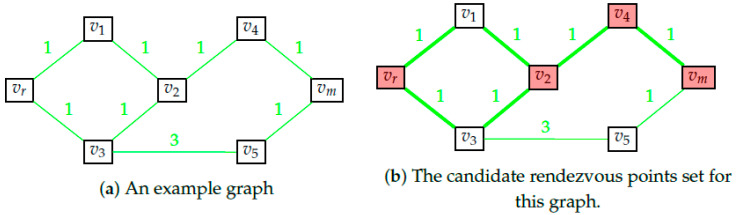
Illustration of candidate rendezvous set. As an illustrative example on obtaining the candidate rendezvous set, consider the graph in (**a**). For the given edge-weights, we have two shortest paths from *v_r_* to *v_m_*, one along*v_r_* − *v*_1_ − *v*_2_ − *v*_4_ − *v_m_* and one along *v_r_* − *v*_3_ − *v*_2_ − *v*_4_ − *v_m_*. The set of points that lie on every shortest path is highlighted in red in (**b**). Thus for this graph $X_{m}$ = {*v_r_*,*v*_2_,*v*_4_,*v_m_*}.

By definition, irrespective of the actual shortest path taken by the rescuee, she will necessarily pass through every point in the candidate rendezvous points set. Figure 2 provides an example graph and highlights the candidate rendezvous set for this graph. As the name suggests, this is the set of points that the rescuer will consider as potential points to rendezvous with the rescuee at. We make an additional assumption on the behavior of the rescuee.

**Assumption** **5.** *The rescuee chooses one path at random, with uniform probability over all the paths in $G_{m}$ to move towards the indicated target $v_{m}$. Unless intercepted by the rescuer at any point in the path, the rescuee stops only once she reaches the indicated target $v_{m}$ and continues to wait there.*

### 2.2. Rescuer Optimal Policy

When deciding which message *m* to send and which node $v_{x}\in X_{m}$ to use as the rendezvous point, the rescuer must take its action keeping the best interests of the rescuee in mind. At the same time, it must also ensure it is passing through regions with low path cost (e.g., ensuring flight path in a relatively safe environment). Accordingly, given a message *m* and meeting point $v_{x}$ the rescuer’s cost is defined as

(4) $$k_{1}\phi_{R}\left(P_{R}^{x}\right)+k_{2}\phi_{r}\left(P_{r}^{x}\right),$$

where $k_{1},k_{2}$ are weights and $P_{R}^{x}\in P_{v_{R}\to v_{x}}$ (respectively, $P_{r}^{x}\in P_{v_{r}\to v_{x}}$) is the splice of path $P_{R}$ to $v_{m}$ joining $v_{R}$ to $v_{x}$ (respectively, of $P_{r}$ joining $v_{r}$ to $v_{x}$). Note here that although the message sent by the rescuer indicates the rescuee to move to either target goals, it is in essence planning to meet the rescuee at one of the candidate rendezvous points (which, in general, may not be the target goal itself). Accordingly, the *cost to rescue* is evaluated as the cost to reach this point. The chosen $v_{x}$ and the corresponding paths $P_{r}^{x}$ and $P_{R}^{x}$ must satisfy the constraint

(5) $$\left(\frac{\phi_{R}\left(P_{R}^{x}\right)}{V_{R}}-\frac{\phi_{r}\left(P_{r}^{x}\right)}{V_{r}}\right){𝟙}_{v_{x}\neq v_{m}}\leq 0,$$

where $V_{R}$ and $V_{r}$ are the constant velocities of the rescuer and rescuee, respectively. The first two terms in the left hand side of (5) can be interpreted as the time taken by the rescuer and rescuee to reach the chosen rendezvous node $v_{x}$, respectively. The third term in the constraint is an indicator variable that takes the value 1 if the chosen rendezvous node is not terminal, and 0 if it is. This constraint indicates that the rescuer must reach the rendezvous point before the rescuee for any non-terminal rendezvous point. It can be observed that this constraint is in line with our Assumption 5 in allowing for a successful rendezvous. We will call any node $v_{x}$ satisfying (5) as a *feasible rendezvous point*.

Defining the ratio of velocities $\frac{V_{R}}{V_{r}}≜k_{v}$ we can re-write the optimization problem to be solved by the rescuer as

(6)minm∈Mminvx∈Xmk1ϕR*(vx)+k2ϕr*(vx)(7)S.T.(ϕR*(vx)−kvϕr*(vx))𝟙vx≠vm≤0(8)where,ϕR*(vx)≜minP∈PvR→vxϕR(P)(9)ϕr*(vx)≜minP∈Pvr→vxϕr(P).

It is important to note that Equation (6) arises out of the Stackelberg framework we laid out for our problem. Equation (9) is a result of the best response of the follower (rescuee) to the signal sent by the leader (rescuer); it arises from our assumption that the rescuee takes shortest paths to the indicated goal and, thus, by principle of optimality, also takes the shortest path to any $v_{x}\in X_{m}$. So Equation (6) is the optimization to be solved by the leader (rescuer) assuming that the follower (rescuee) is playing her best response.

Both (8) and (9) are once again the shortest path problems on a graph and we can solve their equivalent linear problem formulations instead. For any rendezvous point $v_{x}\in X_{m}$ we can re-write (9) as the equivalent linear program (LP),

(10) $$\min_{x_{ij}\geq 0}\sum_{ij\in E}w_{ij}^{r}x_{ij}$$

(11) $$S.T.\;\forall i\;\sum_{j}x_{ij}-\sum_{j}x_{ji}=\left\{\begin{array}{cc} 1 & i=v_{r}\\ -1 & i=v_{x}\\ 0 & \mathrm{otherwise}. \end{array}\right.$$

A similar form can be obtained for (8) by replacing the superscript *r* in (10) with *R*. As indicated in Section 2.1 we can solve the linear programs described above using either simplex methods or by implementing the Dijkstra’s algorithm (DA). Having presented the approach to solve this optimization problem for any node in $X_{m}$, the constrained optimization problem in (6) can be solved by a search over the finite non-empty sets M and $X_{m}$.

## 3. Sensitivity of Optimal Policy to Uncertainty

In the preceding section, we assumed that the topology over the rescue terrain was precisely known to the rescuer. Specifically, the edge weights $w_{ij}^{r}$ and $w_{ij}^{R}$ were assumed to be constant and known to the rescuer. Although it may be reasonable to have some estimate on the nature of the rescue environment (say, from maps and weather forecasts) this estimate is typically uncertain. Another assumption made in the preceding section was that the velocities of the rescuee and the rescuer were known constants. We will see below in Section 3.1 that small changes in the velocity ratio ($k_{v}$) can alter the optimal signaling policy strongly.

Another important assumption we made was related to the human path planning model for the rescuee. Specifically, we assumed that the rescuee is taking shortest paths over the rescue topology faced by her. However, there can be two reasons why such an assumption might be violated.

1. It may be the case that the rescuee may have some uncertainty about the terrain topology surrounding her and thus, may be planning for a path over an incorrect set of edge-weights $w_{ij}^{r}$;
2. Or it is possible that the rescuee simply does not compute shortest paths even with complete knowledge of edge-weights. This may arise either because the rescuee does not have the computational capability to compute shortest paths, or because human path planning is not sufficiently captured by path cost minimization the way we model it.

To arrive at a solution to this problem it is useful to take a step back and look at some empirical studies on human path planning. We postpone further discussion on this to Section 3.3 and begin instead by looking at the other two issues we highlighted above.

### 3.1. Uncertainty in $k_{v}$

**Figure 3:**
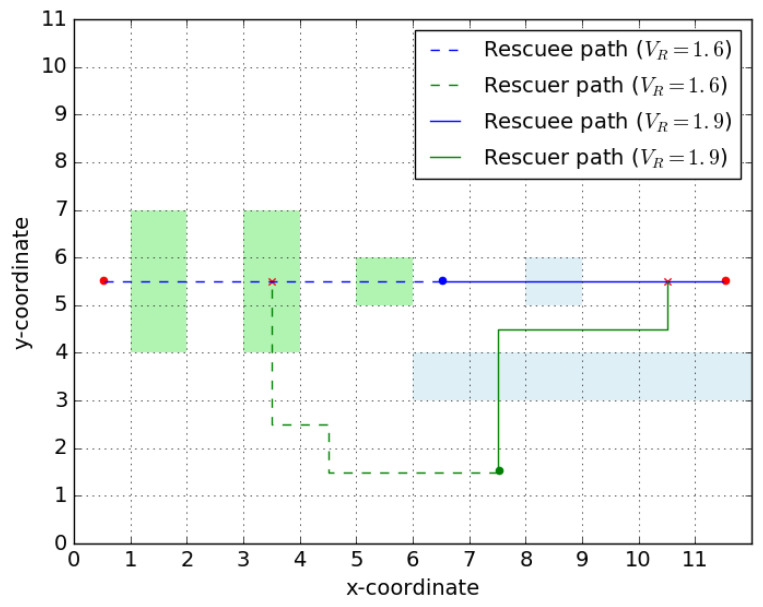
Optimal signal switching with velocity change. Rendezvous trajectories when $k_{v}=1.6$ and $k_{v}=1.9$.

Consider the rescue topology as pictured in Figure 3. The illustration assumes the existence of just two messages M={L,R}. Table 1 presents the optimal signal $m_{opt}$ and the cost to rescuer $U_{R}$ in sending that signal (and performing the subsequent rendezvous) for various values of the velocity ratio $k_{v}$.

We see that the optimal signal to be sent switches multiple times with an increase in $k_{v}$. This sensitivity can be explained as follows. Without loss of generality we assume that rescuee velocity ($V_{r}$) is constant and rescuer velocity ($V_{R}$) is increasing with $k_{v}$. Hills (green shading) take a longer time for rescuee to traverse, and, thus, give more time for the rescuer to rendezvous with her here. However, once the rescuee has traversed the hill and is moving through a region of low cost in between the ‘hill ranges’, she quickly passes through it, getting out of range of the rescuer quickly. Therefore, in the carefully designed scenario presented in Figure 3, the rescuer prefers to send the rescuee towards the ‘left’ plain if the feasible rendezvous point is among these ‘hills’. As the velocity of the rescuer increases, it can reach any point on the path of the rescuee quicker and reduce the cost $U_{R}$ by performing an earlier rendezvous.

For a small grid size like that in Figure 3 we obtained 4 switches in the optimal message to be sent. It can be shown that for every M∈N, there exists some minimum dimension *N* for the grid (N×N) and some topology over the grid, such that the number of switches is greater than *M* (Hebbar [15]).

We showed that small changes in the velocity of the players can strongly affect the outcome of the optimal signaling policy. In our scenario, it might not always be possible for the rescuer to know the exact velocity of the rescuee. Thus, there is a need for a signaling policy that is robust to uncertainty in velocity of players. Section 4.5 presents the robust counter part to (6) and (7) when the velocities of the rescuer and the rescuee are uncertain.

### 3.2. Uncertainty in Edge-Weights $\left\{w_{ij}^{r}\right\}$ and $\left\{w_{ij}^{R}\right\}$

Several difficulties arise if the rescuee’s edge weights $w_{ij}^{r}$ are not known to the rescuer with certainty. First, the rescuer cannot determine the rescuee’s exact set of shortest paths and, a fortiori, the candidate rendezvous points sets $X_{m}$’s. This, in turn, affects the rescuer’s ability to determine if and where a rendezvous can occur. In addition, even if it knew for sure that a given node is visited by the rescuee, the rescuer would be uncertain as to the cost of the path taken by the rescuee, thus making it challenging to evaluate its own actions according to (6) and (7).

In order to address these issues, we first introduce the notion of robust candidate rendezvous set in Section 4, which contains nodes that the rescuee will always traverse and, as we prove, can be computed efficiently by the rescuer. Next, we introduce robust counterparts to (6) and (7) which allow the rescuer to compute the optimal message in the presence of uncertainty in the rescuee’s weights.

### 3.3. Uncertainty in Rescuee’s Path Planning

In Section 2 above, we assumed that the rescuee was taking shortest paths over the rescue topology. However we are now interested in the performance of our signaling approach when the rescuee is planning her path using either ϵ-shortest path (ESP) or vector based navigation (VBN).

#### 3.3.1. Vector Based Navigation (VBN)

**Figure 4:**
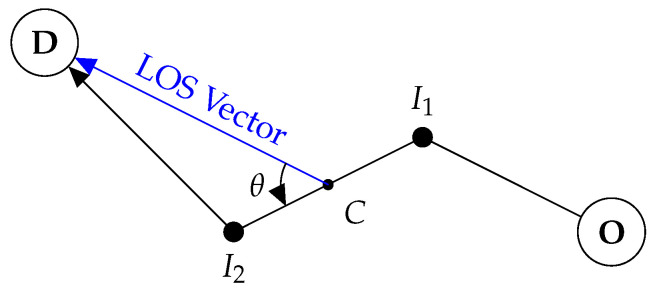
Illustration of LOS (Line-of-Sight) vector from a path segment. In VBN, the cost of traversing the path segment $I_{1}I_{2}$ depends not just on the edge-weight along this edge, but also the angle θ made by the segment with the LOS vector from the mid-point *C* of the segment to the destination.

In a VBN model, the cost of traversing a path segment depends on the angle made by the path segment with the the line-of-sight (LOS) vector to the destination (See Figure 4). Motivated by Weber–Fechner Law of Just Noticeable Difference, Bongiorno et al. [13] picks a log-normal distribution model for the costs associated with a path segment. Inspired by this approach we will model the the cost-to-traverse a path segment between nodes *i* and *j* with cost $w_{ij}$ and making angle θ∈[−π,π] with LOS as

$$c_{ij}\left(\theta \right)=w_{ij}\times \left(1+\exp\left(K_{los}|\theta |\right)\right).$$

For a given origin-destination pair, the cost of any path segment is a fixed constant and the rescuee tries to minimize the path cost of going from the origin to the destination. In other words, the rescuee is simply finding shortest paths over a graph with augmented edge-weights $c_{ij}$ instead of $w_{ij}$.

**Figure 5:**
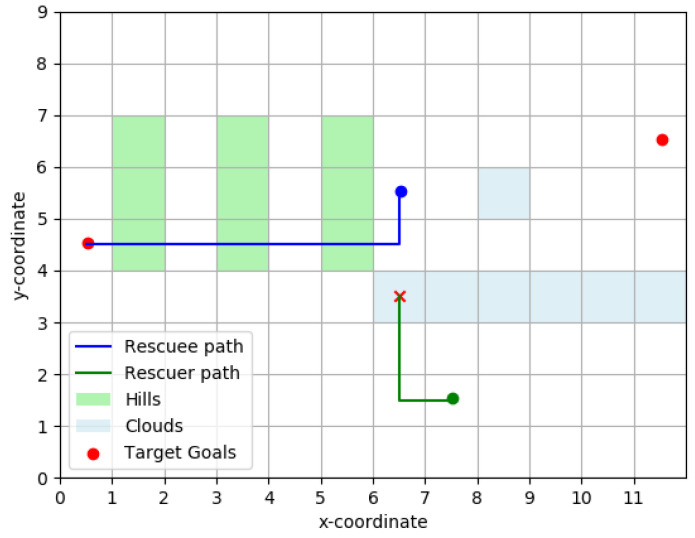
Strips layout $K_{los}=1$. For the rescuee, the green shaded cells have a cost-to-traverse randomly sampled from [2.5,3] and all other cells have a cost-to-traverse picked from [1,1.5]. The topology faced by the rescuee in going left resembles a parallel range of hills with valleys between them and we will name this general layout ‘strips’ for convenience in referring to. The rescuer planned to meet in the grid-square (6,3). Here a grid square is denoted by co-ordinates of bottom left corner.

Figure 5 shows a particular topology layout for our rescue scenario. The rescuee takes an LOS path to the goal, while the rescuer plans for a rendezvous assuming the rescuee is taking shortest paths over the topology. If the rescuee had indeed taken shortest path to the signaled goal, she would have passed through the rendezvous point picked by the rescuer.

#### 3.3.2. ϵ-Shortest Paths (ESP)

**Figure 6:**
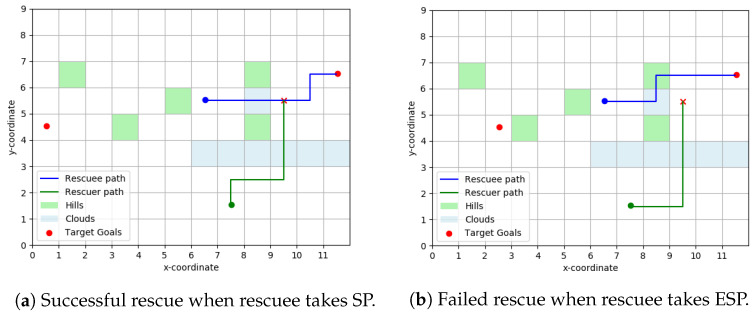
Scatter Layout. For the rescuee, the green shaded cells have a cost-to-traverse randomly sampled from [2.5, 3] and all other cells have a cost-to-traverse picked from [1, 1.5]. We label this terrain topology as ‘scatter’ layout as the ‘hills’ are scattered around. The rescuer planned to rendezvous at (9, 5).

Relaxing Assumption 5, we will assume now that the rescuee computes the set of ϵ-shortest paths between any origin-destination pair and picks one at random from this set. Naturally, ϵ-shortest path set is a superset of shortest paths set. It is easy to create scenarios where the rescuer, assuming human takes shortest path, fails to rendezvous with the rescuee taking an ϵ-shortest path. One such situation is illustrated in Figure 6b where ϵ is taken to be 0.2. The path taken by the rescuee here has a path length less than 1.2 times the shortest paths length from her initial position to the right target goal.

As expected, our algorithm performs well when the rescuee is indeed path-planning using the shortest path (SP) approach but fails to rendezvous with the her when she takes the ϵ−shortest path shown in Figure 6b.

Having highlighted the issues that may arise when Assumptions 1–5 are invalidated we will now seek to arrive at signaling policies that are robust to the issues discussed in this section. In Section 4 we present an approach that accounts for both travel cost and rescuer velocity uncertainty. Such an approach is then robust to the issues underscored in Section 3.1 and Section 3.2. In Section 5, we present an alternate approach that exploits the distributional information we may have over the edge-costs in the rescue topology graph to reduce the conservativeness of the robust approach. This approach, as we will see, is computationally expensive but can also be modified to account for the alternate models of human path planning discussed in Section 3.3.

## 4. Robust Signaling Approach

From now on, we assume that the edge weights $w_{ij}^{r}$ and $w_{ij}^{R}$ are unknown but bounded: ${\underline{w}}_{ij}^{r}\leq w_{ij}^{r}\leq {\bar{w}}_{ij}^{r}$ and ${\underline{w}}_{ij}^{R}\leq w_{ij}^{R}\leq {\bar{w}}_{ij}^{R}$∀ij∈E. We designate the Cartesian product $\Pi_{ij\in E}\left[{\underline{w}}_{ij}^{r},{\bar{w}}_{ij}^{r}\right]$ as $\Omega^{r}$ and $\Pi_{ij\in E}\left[{\underline{w}}_{ij}^{R},{\bar{w}}_{ij}^{R}\right]$ as $\Omega^{R}$ and any arbitrary element from this set is denoted by $w^{r}$ and $w^{R}$, respectively. The rescuee has complete knowledge of the realized edge-weights. The rescuer has knowledge about the supports $\Omega^{r}$ and $\Omega^{R}$ but does not know the true realization of these edge-weights. Effectively, in this section we are doing away with Assumption 3.

We make an observation that in the analysis presented in Section 2 the edge weights only show up when we seek to find the shortest paths over the graph. There can be multiple approaches in arriving at the shortest path in a graph with uncertain weights. Treating this uncertainty as stochastic, Dantzig [14] replaced the edge weights of an uncertain graph with their expected values and solved the resulting shortest path problem with certain weights. The problem with this approach is that there exists a non-zero, and often large probability that the resulting shortest path is strongly sub-optimal. Sigal et al. [16] and Frank [17] proposed methods to maximize the probability that a certain path realizes the least weight. Such probabilistic approaches are reasonable when we are running the uncertain scenario over multiple iterations and seek only to minimize the expected cost over the runs. However, in success critical problems such as our rescue scenario, we may wish to be completely risk-averse. A natural step forward is then to consider a robust optimal approach in designing our signaling policy.

We presented the linear program formulation of the shortest path problem in Section 2. The same problem can also be presented as an integer programming problem, with each $x_{ij}^{r},x_{ij}^{R}\in \left\{0,1\right\}$ (Dantzig [14]). Efficient ways to compute the robust optimal solution for this formulation were presented by Bertsimas and Sim [18] assuming an upper bound on the number of edge-weights that are uncertain. We will work with the more general (and simpler) scenario where we assume all edge-weights are uncertain. In our work, we use the notions of a robust counterpart to an optimization problem as presented by Ben-Tal et al. [19].

### 4.1. Robust Optimal Candidate Rendezvous Set

Before the rescuer can determine which message to send in presence of uncertainty (i.e., the message *m* such that the worst-case value of cost (4) is minimized when $w^{r}$ and $w^{R}$ belongs to $\Omega^{r}$ and $\Omega^{R}$), it is necessary to evaluate the set of possible rendezvous points (if, for nothing else, to appropriately compute that worst-case value). This naturally leads us to introduce the following

**Definition** **2.** *Let $X_{m}^{w^{r}}$ be the candidate rendezvous points set as defined in Definition 1 for the rescuee’s edge weights $w^{r}$. The* robust candidate rendezvous *set is defined as*

(12) $${\hat{X}}_{m}=\underset{w^{r}\in \Omega^{r}}{⋂}X_{m}^{w^{r}}.$$

**Figure 7:**
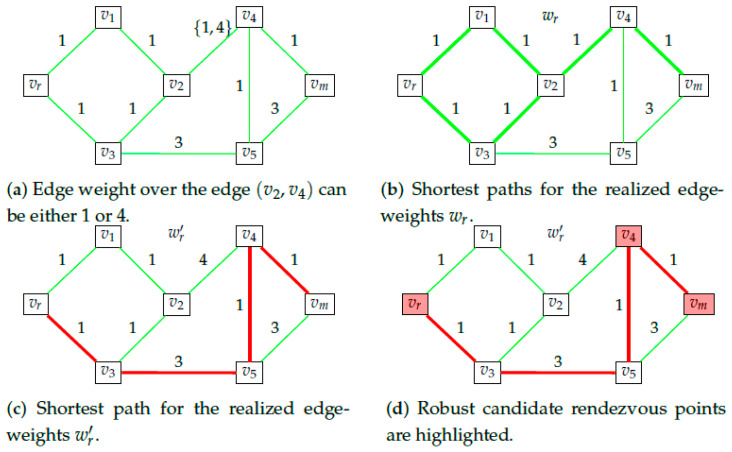
Illustration of robust candidate rendezvous set. (**a**) shows the graph in consideration. We see that the edge weights over all edges except one are constants. (**b**,**c**) show the shortest paths over the graph for two different realizations of edge-weights. (**d**) highlights the set of robust candidate rendezvous points. So we have $X_{m}$ = {*v_r_*,*v*_4_,*v_m_*}.

In words, the *robust candidate rendezvous set* is the set of nodes that lie on the shortest path for every possible set of edge-weights. Figure 7 gives an illustrative example where this set is computed explicitly. Clearly, ${\hat{X}}_{m}$ cannot be readily computed from (12) because it involves a countable intersection of sets (each of which is tractable, as explained in Section 2). Fortunately, it is possible to state the following,

**Proposition** **1.** *Algorithm 1 presented below terminates, computes the set ${\hat{X}}_{m}$ and runs in $O(|V{|}^{3})$.*

A proof of correctness for this algorithm is provided in Appendix A. In a graph with positive edge-weights, all equivalent shortest paths $P^{*}$ can be obtained using a minor modification of the Dijkstra’s algorithm. If the implementation of Dijkstra’s algorithm runs in $O(|V{|}^{2})$, then Algorithm 1 presented above runs in $O(|V{|}^{3})$. This polynomial time-complexity for Algorithm 1 preserves the efficiency of our approach in finding the robust optimal signaling policy.
**Algorithm 1.** Algorithm to find the robust candidate rendezvous points**Result**: Obtain ${\hat{X}}_{m}$ Set edge weights $\left\{w_{ij}^{r1}\right\}=\left\{{\underline{w}}_{ij}^{r}\right\}$; Find the graph of shortest path $G_{m}^{*1}=\left(V_{m}^{*1},E_{m}^{*1}\right)$ and the corresponding set of paths ${\left\{P^{*}\right\}}^{1}$; Initialize ${\hat{X}}_{m}^{1}=\{v:v\in P\;\forall P\in {\left\{P^{*}\right\}}^{1}$; Set $F^{1}=\left\{ij:w_{ij}^{r1}={\underline{w}}_{ij}^{r},ij\in G_{m}^{*1}\right\}$; Set k=1; 
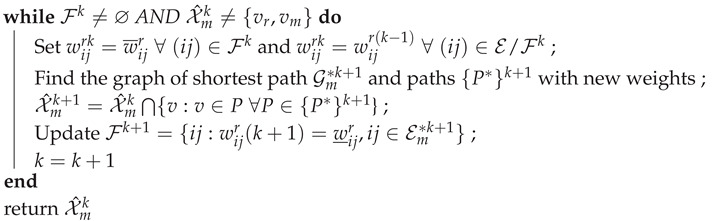


### 4.2. Robust Optimal Signal Computation

We make an assumption on the ratio of velocities $k_{v}$ for the remainder of our work.

**Assumption** **6.** *Let*

$$w_{\max}^{R}≜\max_{ij}{\bar{w}}_{ij}^{R}$$

$$\mathrm{and}\;w_{\min}^{r}≜\min_{ij}{\underline{w}}_{ij}^{r}.$$

*Then, we assume a lower bound on the ratio of velocities,*

(13) $$k_{v}≜\frac{V_{R}}{V_{r}}\geq \frac{w_{\max}^{R}}{w_{\min}^{r}}.$$

This assumption formalizes the notion that the rescuer can move faster on any part of the terrain than the rescuee. It is worth noting that this assumption alone does not guarantee the existence of non-terminal rendezvous point. It merely implies that on any given path on G, the rescuer takes less time than the rescuee. Thus, if the rescuee was substantially closer to the target goal than the rescuer, the larger speed of the rescuer may still not help it reach some intermediate node on the rescuee’s path.

**Definition** **3.** *For any two nodes $v_{m},v_{n}$ in the graph G we define a partial ordering ‘≤’ as*

$$v_{m}\leq v_{n}\;\mathrm{if}\;\phi_{r}^{*}\left(v_{m}\right)\leq \phi_{r}^{*}\left(v_{n}\right),$$

with $\phi_{r}^{*}$ defined in (9).

Without loss of generality, we can list all the nodes in ${\hat{X}}_{m}$ in increasing order as, $v_{r}=v_{x,1}\leq v_{x,2}\leq \cdots \leq v_{x,L}=v_{m}$, where $L=|{\hat{X}}_{m}|$. Assumption 6 leads us to,

**Proposition** **2.** *For any m,n, such that 1≤m<n≤L and for any realization $w^{r}\in \Omega^{r},w^{R}\in \Omega^{R}$ we have*

$$\begin{array}{c} \phi_{R}^{*}\left(v_{x,m}\right)-k_{v}\phi_{r}^{*}\left(v_{x,m}\right)\leq 0\Rightarrow \phi_{R}^{*}\left(v_{x,n}\right)-k_{v}\phi_{r}^{*}\left(v_{x,n}\right)\leq 0, \end{array}$$

with $\phi_{R}^{*}$ and $\phi_{r}^{*}$ are given by (8) and (9), respectively.

Proof for this proposition can be found in Appendix B. We basically showed that if a node is a robust *feasible* rendezvous point then all nodes in ${\hat{X}}_{m}$ succeeding (ordered by Definition 3) this point are also robust *feasible* rendezvous points. From this, it is easy to see

**Corollary** **1.** *If there exists at least one robust feasible rendezvous point then necessarily $v_{m}$ is also a robust feasible rendezvous point.*

We are now equipped to analyze the problem of finding the optimal signaling policy when faced with uncertain path costs. To do so, we can first break our problem into two cases.

- There always exists at least one robust feasible rendezvous point for all possible edge-weights;
- No robust feasible rendezvous point for some $w^{R},w^{r}$.

By Corollary 1 it suffices to check whether $v_{m}$ is a robust feasible rendezvous point to verify which of the two cases is at hand.

### 4.3. Case I: At-Least One Robust Feasible Rendezvous Point

In this case, for any $w^{r}\in \Omega^{r}$ and $w^{R}\in \Omega^{R}$ the constraint in (7) for a feasible rendezvous point $v_{x}$ simplifies to the form,

(14) $$\phi_{R}^{*}\left(v_{x}\right)-k_{v}\phi_{r}^{*}\left(v_{x}\right)\leq 0.$$

For any candidate rendezvous point $v_{x}\in {\hat{X}}_{m}$ we define $\phi_{R,\max}^{*}\left(v_{x}\right)$ and $\phi_{r,\min}^{*}\left(v_{x}\right)$ as,

(15) $$\begin{array}{cc} \phi_{R,\max}^{*}\left(v_{x}\right) & ≜\min_{x_{ij}\geq 0}\sum_{ij\in E}{\bar{w}}_{ij}^{R}x_{ij} \end{array}$$

$$\begin{array}{cc} S.T.\;\forall i\;\sum_{j}x_{ij}-\sum_{j}x_{ji} & =\left\{\begin{array}{cc} 1 & i=v_{R}\\ -1 & i=v_{x}\\ 0 & \mathrm{otherwise}. \end{array}\right. \end{array}$$

(16) $$\begin{array}{cc} \mathrm{Therefore},\;\phi_{r,\min}^{*}\left(v_{x}\right) & ≜\min_{x_{ij}\geq 0}\sum_{ij\in E}{\underline{w}}_{ij}^{r}x_{ij} \end{array}$$

$$\begin{array}{cc} S.T.\;\forall i\;\sum_{j}x_{ij}-\sum_{j}x_{ji} & =\left\{\begin{array}{cc} 1 & i=v_{r}\\ -1 & i=v_{x}\\ 0 & \mathrm{otherwise}. \end{array}\right. \end{array}$$

With these definitions in hand, we can state the following

**Proposition** **3.** *Under the conditions on this subsection, the message minimizing worst-case cost for the rescuer when $w^{r}$ varies in $\Omega^{r}$ and $w^{R}$ varies in $\Omega^{R}$ is a solution to the program*

(17) $$\min_{m\in M}\min_{v_{x}\in X_{m}}k_{1}\phi_{R,\max}^{*}\left(v_{x}\right)+k_{2}\phi_{r,\max}^{*}\left(v_{x}\right)$$

*subject to the constraint*

(18) $$\phi_{R,\max}^{*}\left(v_{x}\right)-k_{v}\phi_{r,\min}^{*}\left(v_{x}\right)\leq 0,$$

where $\phi_{r,\max}^{*}\left(v_{x}\right)$ can be obtained by replacing ${\underline{w}}_{ij}^{r}$ in (16) with ${\bar{w}}_{ij}^{r}$.

### 4.4. Case II: No Robust Feasible Rendezvous Point

In the scenario where we have no robust feasible rendezvous point, the only way to guarantee a successful rendezvous is by meeting the rescuee at the target goal node $v_{m}$ corresponding to the message *m*. Thus, the robust counterpart to (6) in this case is simply

(19) $$\min_{m\in M}k_{1}\phi_{R,\max}^{*}\left(v_{m}\right)+k_{2}\phi_{r,\max}^{*}\left(v_{m}\right),$$

where $\phi_{R,\max}^{*}\left(v_{m}\right)$ and $\phi_{r,\max}^{*}\left(v_{m}\right)$ is obtained as we did for case I.

### 4.5. Robustness to Velocity Variation

In this section, we briefly discuss the robustification of our signaling policy from Section 2 to uncertainties in the velocities of our agents (or equivalently, violation of Assumption 4). We will encompass the variation in velocity of the rescuer and the rescuee in variations of the parameter $k_{v}$. We assume that $k_{v}$ can take on arbitrary values in the interval $\left[{\underline{k}}_{v},{\bar{k}}_{v}\right]$.

The objective function in (6) is unaffected by the value of $k_{v}$. The constraint (5) is affine in $k_{v}$. From the results of Ben-Tal et al. [19], the robust counterpart to the optimization in (6) subject to (5) is obtained as

(20)minm∈Mminvx∈Xmk1ϕR*(vx)+k2ϕr*(vx)(21)S.T.(ϕR*(vx)−k_vϕr*(vx))𝟙vx≠vm≤0,

with $\phi_{R}^{*}$ and $\phi_{r}^{*}$ given by (8) and (9), respectively. The edge weights $w^{r}$ and $w^{R}$ are assumed fixed and known above. The robustification of the optimization in Section 2 with respect to the edge weights and the parameter $k_{v}$ can be performed independently. For the subsequent robustification of the constraint in (21) with respect to edge-weight uncertainty, we only need ${\underline{k}}_{v}$ to satisfy the constraint in Assumption 6.

### 4.6. Simulation Example

**Figure 8:**
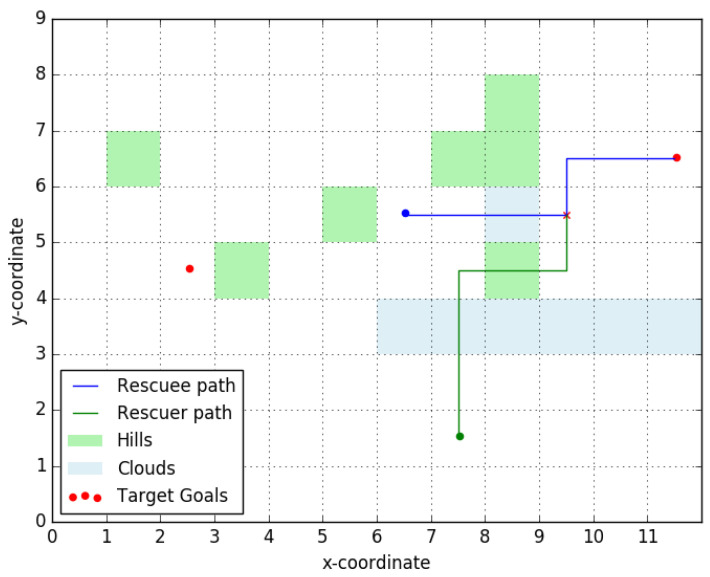
Robust optimal signaling policy in a simulated run. Clouds (blue shading) act as ‘high edge weight’ regions to the UAV and hills (green shading) act as ‘high edge weight’ regions to the rescuee. The optimal signal sent out was indicating the right target goal.

We now present the results of a simple simulation on a carefully designed rescue topology to highlight some features of the approach designed in above. The parameters chosen are $k_{1}=1$, $k_{2}=1$ and $k_{v}=3.1$. All edge weights $w_{ij}^{r}$ and $w_{ij}^{R}$ are unknown to the rescuer and can be of two types. ‘High edge weight’, where each edge weight is a random variable is supported over [2.5,3] and ‘low edge weight’, each supported over [1,1.5]. It can be verified that Assumption 6 holds for these set of edge weights and the velocity ratio $k_{v}$. The topology over the rescue terrain, as well as the path’s travelled by the rescuee and the rescuer are presented in Figure 8.

It can be observed that although the left target goal was spatially closer to both the rescuee and the rescuer, the rescuer signals the rescuee to go towards the right goal. In going right the set $X_{m}$ consists of grid squares (7,6),(8,5),(9,5) (We denote the position of a grid square using the coordinates of its bottom left corner.). In going left there exist no such non-terminal points in the set $X_{m}$. This availability of robust candidate rendezvous points encourages the rescuer to signal going right.

## 5. Stochastic Signaling Approach

In the preceding section, we considered a robust approach in finding an optimal signaling policy for our rescue scenario. Such an approach trades optimality for robustness by designing a policy that only maximizes the rewards in the worst-case scenario. It may often be the case that the probability of the worst cases scenario being realized is very small. It may also be the case that optimal action in the worst cases scenario is strongly sub-optimal in many of the other scenario. It is not difficult to construct a scenario that achieves such sub-optimality in our framework.

**Figure 9:**
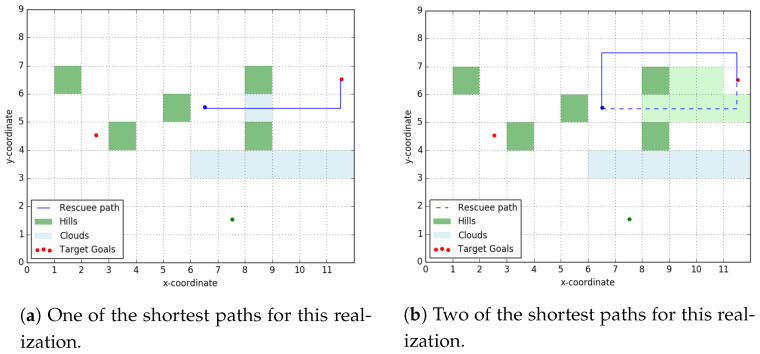
Conservativeness of the robust policy. The dark green hilly regions have a cost-to-traverse of 3 for the rescuee. Each white grid square has a cost to traverse of 1, while in the second figure the light green shaded grid squares have a cost to traverse of 1.5.

Figure 9 shows two realizations of the ‘scatter’ layout introduced in Section 3. We will introduce ‘stochasticity’ in our layout by assuming that the cost-to-traverse over green grid squares in Figure 6b is uniformly distributed over [2.5,3] and over white squares it is uniformly distributed over [1,1.5].

When signaled to go ‘right’, the rescuee will traverse through grid-squares (7,5),(8,5), and (9,5) (squares denoted by coordinate of their bottom-left corner) with probability 1 (Hebbar [15]). So these form the *candidate rendezvous set* with a high probability. However, the *robust signaling policy* developed in Section 4 also accounts for the zero probability scenario shown in Figure 9b, where a shortest path (solid blue line) does not pass through these three points. So the *robust optimal signaling policy* will plan to meet the rescuee at the ‘right’ target goal, when instead, with a probability 1 the rescuer could have rendezvoused with the rescuee at (9,5), as shown in Figure 6a.

This illustration highlights the conservativeness of the signaling policy we designed in the previous section. Additionally, we may often have some distributional information over the edge-weights and the robust optimal signaling policy makes no use of this available information. So with the aim of addressing both these issues and motivated by the discussion above we ask a naturally arising question—‘What is the set of nodes that will lie on all shortest paths with a high probability, when the edge-weights vary stochastically?’

The problem of finding distributional information over path lengths in graphs with stochastic edge-weights is NP-hard (Valiant [20]) and the traditional approach to solving problems from this family is using Monte Carlo methods. Frank [17] proposes the following condition for path optimality: For a specified k, consider the path that maximizes the probability of realizing a weight less than k as the optimal path. Another approach taken by Sigal et al. [16] is to find the path with the greatest probability of realizing the least weight. Their work then assigns ‘path optimality indices’ to each path being considered. Formally, these paths are solutions to

$$\begin{array}{c} P^{*}=\underset{P\in P_{S\;\to \;G}}{arg max}\;\;\prod_{P^{\prime }\;\in P_{S\;\to \;G}/\left\{P\right\}}P\left\{L\left(P\right)\leq L\left(P^{\prime }\right)\right\}. \end{array}$$

Motivated by this idea we will design a similar approach to find the candidate rendezvous points set is a stochastic setting. We will define the notion of ‘node candidacy index’ much like ‘path optimality indices’ and use Monte-Carlo methods to obtain it.

### 5.1. Stochastic Candidate Rendezvous Set

Going forward, we assume that the edge weights $w_{ij}^{r}$ and $w_{ij}^{R}$ are stochastic bounded random variables: ${\underline{w}}_{ij}^{r}\leq w_{ij}^{r}\leq {\bar{w}}_{ij}^{r}$ and ${\underline{w}}_{ij}^{R}\leq w_{ij}^{R}\leq {\bar{w}}_{ij}^{R}$∀ij∈E. As before, we designate the Cartesian product $\Pi_{ij\in E}\left[{\underline{w}}_{ij}^{r},{\bar{w}}_{ij}^{r}\right]$ as $\Omega^{r}$ and $\Pi_{ij\in E}\left[{\underline{w}}_{ij}^{R},{\bar{w}}_{ij}^{R}\right]$ as $\Omega^{R}$. We can then consider the random vectors $w^{r}={\left\{w_{ij}^{r}\right\}}_{ij\in S}$ and $w^{R}={\left\{w_{ij}^{R}\right\}}_{ij\in S}$ over the supports $\Omega^{r}$ and $\Omega^{R}$, respectively. We will refer to probabilities using notation P and *P* will be reserved to denote paths as before.

**Definition** **4.** Let $P_{m}^{*}\left(w^{r}\right)$ denote the shortest path taken by the rescuee to the target goal $v_{m}$ when the realised edge-weights are $w^{r}$. We then define a candidacy index for each node as a real-valued map J:V→R with

$$\begin{array}{c} J\left(v\right)=P(v\in P_{m}^{*}\left(w^{r}\right)). \end{array}$$

Definition 4 captures the essence of the answer to the question we posed earlier in this section. Any node with a ‘high’ candidacy index will lie on a shortest path between the rescuee’s initial location and the target goal with a high probability. Then, in this ‘stochastic approach’, a natural way to define some form of ‘candidate rendezvous set’ would be to consider all nodes with candidacy index above a certain threshold. Mathematically, we want to define a set of the form

(22) $$\left\{v:J(v)>1-\gamma \right\}$$

for some 1>γ>0. However, computing such a set is challenging as there is no easy way for us to compute the node candidacy index. Therefore, we turn to Monte Carlo methods.

However, before we do so, it must be noted that the ‘shortest path’ as stated in the Definition 4 above may not be unique. In Assumption 5, we noted that when there are multiple equivalent shortest paths the rescuee picks one at random with a uniform probability over all paths. To capture this additional stochasticity in the choice of shortest path, we will treat $P_{m}^{*}\left(w^{r}\right)$ as a random vector in itself. Recall that in the LP formulation for the shortest path problem we represent any path as an *m*-dimensional vector with boolean elements. So it is meaningful to talk about a path as a random vector. We then rewrite the *candidacy index* as an expectation as

$$\begin{array}{c} J\left(v\right)=P\left(v\in P_{m}^{*}\left(w^{r}\right)\right)=E_{w^{r},P_{m}^{*}}\left[{𝟙}_{v\in P_{m}^{*}\left(w^{r}\right)}\right], \end{array}$$

where the expectation is taken with respect to both the distribution over the edge-weights $w^{r}$ and the uniform distribution over the choice of shortest path $P_{m}^{*}$. We then present the following

**Definition** **5.** Let $P_{m}^{*}\left(w^{r}\right)$ be the set of all shortest paths from $v_{r}$ to $v_{m}$ when the edge-weight vector is $w^{r}$. We define the candidacy index estimator

$$\begin{array}{c} g\left(v,w^{r}\right)=\frac{|P:v\in P,P\in P_{m}^{*}\left(w^{r}\right)|}{|P_{m}^{*}\left(w^{r}\right)|}. \end{array}$$

We can then state the following proposition,

**Proposition** **4.** $$\begin{array}{c} E_{w^{r}}\left[g\left(v,w^{r}\right)\right]=J\left(v\right). \end{array}$$

Proof for this proposition is presented in Appendix B. We are now fully equipped to apply Monte-Carlo methods to our problem to approximate the *candidacy index* as,

$$\begin{array}{c} J\left(v\right)\approx \tilde{J}\left(v\right)≜\frac{1}{K}\sum_{i=1}^{K}g\left(v,w_{i}^{r}\right) \end{array}$$

where *K* is a free parameter determining the number of samples we take to approximate J(v) and $w_{i}^{r}$ is the $i^{th}$ sampled edge-weight vector. Motivated by (22), we introduce the following

**Definition** **6.** *For some $1>\bar{\gamma }>0$, we define the stochastic candidate rendezvous points set as*

$$\begin{array}{c} {\tilde{X}}_{m}=\left\{v\in V:\tilde{J}\left(v\right)>1-\bar{\gamma }\right\}. \end{array}$$

Now to find this stochastic candidate rendezvous set we need to compute the candidacy indices for all nodes. We know that the shortest paths can be computed using Dijkstra’s algorithm in O(|E|+|V|log|V|). The primary computational complexity in obtaining the candidacy index then arises from the need for a large number of samples. So the question now remains: “What is the minimum number of samples *K* to be picked such that the candidacy index estimate $\tilde{J}\left(v\right)$ is a ‘good’ approximation of the true candidacy index J(v)?” Requiring a small number of samples here is key in our ability to compute the final optimal signaling policy on board the UAV. It can be shown that, for a given ε and δ, the minimum number of samples *K* required in computation of $\tilde{J}\left(v\right)$ to achieve

$$\begin{array}{c} P(|\tilde{J}\left(v\right)-J\left(v\right)|\geq \varepsilon )\leq \delta \end{array}$$

is of the order of $O\left(-\frac{\log\delta }{\varepsilon^{2}}\right)$ (Hebbar [15]). We see that it is much more computationally expensive to desire a smaller ϵ than a smaller δ.

We are interested in obtaining nodes with *true candidacy index*, J(v), above threshold 1−γ. To ensure this we can pick nodes with *estimated candidacy index*, $\tilde{J}\left(v\right)$ above threshold $1-\bar{\gamma }$ where $\bar{\gamma }<\gamma -\varepsilon$. For such a choice of $\bar{\gamma }$, it is easy to show that

$$\begin{array}{c} \forall v\in {\tilde{X}}_{m}\;P\left(J\left(v\right)<1-\gamma \right)<\delta . \end{array}$$

So we see that we have some degree of freedom in our choice of ϵ through the choice in $\bar{\gamma }$, although choosing a smaller $\bar{\gamma }$ will lead to more conservativeness. However this also means that we are free to choose a slightly larger ϵ and reduce the computational complexity of the Monte Carlo method.

### 5.2. Stochastic Optimal Signaling Policy

In the preceding section we computed the stochastic candidate rendezvous set, i.e., ‘the set of points that lie on some shortest path with a high probability when the edge-weights are varied stochastically.’ We now present a modified version of the robust approach developed in Section 4 where we use the *stochastic candidate rendezvous set* in lieu of the *robust candidate rendezvous set*. We propose the following optimization to be solved by the rescuer to arrive at the optimal signal to be sent when non-terminal feasible points exist among our candidate set.

**Proposition** **5.** *Any stochastic feasible rendezvous point $v_{x}\in {\tilde{X}}_{m}$ for (14) satisfies*

(23) $$\phi_{R,\max}^{*}\left(v_{x}\right)-k_{v}\phi_{r,\min}^{*}\left(v_{x}\right)\leq 0,$$

where $\phi_{R,\max}^{*}\left(v_{x}\right)$ and $\phi_{r,\min}^{*}\left(v_{x}\right)$ are obtained as (15) and (16), respectively. The stochastic robust counterpart to the optimization problem presented in (6) is then given by

(24) $$\min_{m\in M}\min_{v_{x}\in {\tilde{X}}_{m}}k_{1}\phi_{R,\max}^{*}\left(v_{x}\right)+k_{2}\phi_{r,\max}^{*}\left(v_{x}\right)$$

subject to (23), where $\phi_{r,\max}^{*}\left(v_{x}\right)$ can be obtained by replacing ${\underline{w}}_{ij}^{r}$ in (16) with ${\bar{w}}_{ij}^{r}$. □

Once again in the case where there are no feasible rendezvous points the optimal signal to be sent can be arrived at by comparing the cost to rendezvous at the terminal goals.

Note here that (23) is not evaluated in a probabilistic manner. We only use the knowledge of distribution over edge-weights in evaluating the *stochastic candidate rendezvous set*. However, we then evaluate feasibility using the robust method developed in Section 4. Assessing the feasibility of a particular *candidate rendezvous point* requires us to compute the shortest path to this point for both the rescuer and the rescuee. The number of *candidate rendezvous points* is of the order O(|V|) and evaluating this feasibility for every point, for each sampled edge-weight vector, would require us to run the shortest paths algorithm O(|V|) times. In comparison, obtaining the candidate rendezvous set for each sampled edge-weight vector requires only one run of the shortest paths algorithm. So with the aim of keeping our computational complexity low, we employ the Monte-Carlo inspired approach only to arrive at the *candidate rendezvous points set* and stick to the robust method to evaluate feasibility.

### 5.3. Stochastic ϵ-Optimal Signaling Policy

In Section 3.3 we showed that if the rescuee does not pick shortest paths in its path planning model, the *optimal signaling policy* developed in Section 2 performs poorly. We also discussed two alternate models of human path planning—ESP and VBN—that may better model rescuee behavior. However, there is vast evidence (Bongiorno et al. [13], Zhu and Levinson [12], and Malleson et al. [21] among others) that irrespective of the true model of human path planning behavior, the final path picked is not far from optimal, i.e., the path picked by humans is usually an ϵ-shortest path. Although the value of ϵ to be picked is a point of contention, accounting for all ϵ-shortest paths in the signaling policy seems like a step in the right direction.

The number of ϵ-shortest simple paths grows exponentially with the number of nodes |V| in a network when ϵ is increased. Of course, in the extreme case where ϵ is large enough that all simple paths over the graph are admissible as ϵ−shortest, the number of simple paths between any two nodes in a the graph can be of the order of O((|V|−2)!). So it is clear that we cannot achieve a general polynomial (in graph parameters) time algorithm to find all ϵ−shortest paths for any arbitrary ϵ.

Byers and Waterman [22] and Naor and Brutlag [23] suggest approaches to compute such ϵ-shortest paths with time complexity scaling linearly with the number of ϵ-shortest paths. An alternate strategy to compute ϵ-shortest paths is to instead compute the *k* shortest paths over the graph. The number *k* is increased until we find the *k* corresponding to the length limit. Eppstein [24] runs a length limited *k* shortest path algorithm to find *k* simple paths with length at most (1+ϵ) times the shortest path. Yen [25] presents an efficient polynomial time $\left(O\left(k|V|(|E|+|V|\log|V|)\right)\right)$ algorithm to compute *k* loopless shortest paths. Eppstein [24] provides an alternate heap-implementation which can obtain the *k* shortest paths in time O(|V|+|E|+k) after a spanning graph has been computed. However, this implementation returns paths with loops. So we would have to compute a lot more than *k*-shortest paths using this method to actually obtain *k*-shortest *loopless* paths. Our implementation runs the Yen’s algorithm.

**Definition** **7.** *Let $P_{m}^{\epsilon }\left(w^{r}\right)$ be the set of all ϵ-shortest paths from $v_{r}$ to $v_{m}$ when the edge-weight vector is $w^{r}$. We define the*ϵ-candidacy index estimator

$$\begin{array}{c} g^{\epsilon }\left(v,w^{r}\right)=\frac{|P:v\in P,P\in P_{m}^{\epsilon }\left(w^{r}\right)|}{|P_{m}^{\epsilon }\left(w^{r}\right)|}. \end{array}$$

Proceeding as before we assume that ϵ-shortest path picked by the rescuee ($P_{m}^{\epsilon }\left(w^{r}\right)$) is a random variable with uniform distribution over the set of all ϵ−shortest paths. We can then define the ϵ-candidacy index as

$$\begin{array}{c} J^{\epsilon }\left(v\right)≜P\left(v\in P_{m}^{\epsilon }\left(w^{r}\right)\right)=E_{w^{r},P_{m}^{\epsilon }}\left[{𝟙}_{v\in P_{m}^{\epsilon }\left(w^{r}\right)}\right]. \end{array}$$

Next, we approximate this ϵ-candidacy index using Monte-Carlo methods as

$$\begin{array}{c} J^{\epsilon }\left(v\right)\approx {\tilde{J}}^{\epsilon }\left(v\right)≜\frac{1}{K}\sum_{i=1}^{K}g^{\epsilon }\left(v,w_{i}^{r}\right). \end{array}$$

For some $1>\tilde{\gamma }>0$, we can then define the ϵ-candidate rendezvous set as

$$\begin{array}{c} {\tilde{X}}_{m}^{\epsilon }=\left\{v\in V:{\tilde{J}}^{\epsilon }\left(v\right)>1-\tilde{\gamma }\right\}. \end{array}$$

We simply use this *ϵ-candidate rendezvous set* (${\tilde{X}}_{m}^{\epsilon }$) in lieu of the *stochastic candidate rendezvous set* (${\tilde{X}}_{m}$) in Proposition 5 to obtain a signaling policy that is relatively more robust to uncertainty in rescuee’s path planning model.

## 6. Results

**Figure 10:**
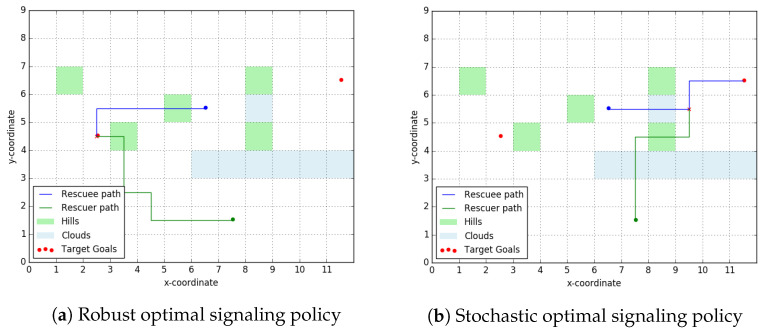
Comparative run of the robust and stochastic signaling policies. Over the same topographical layout we observe that the optimal signal to be sent changes with the robustness criteria we choose. In (**a**), the rescuer employs the completely robust signaling policy and incurs a worst case cost-of-rescue of 19.5. In (**b**) the rescuer incurs a worst case cost-of-rescue of 13. Here, we take *K* = 50 samples and 1 − *γ* = 0.9 as the threshold for admitting a node into the stochastic candidate rendezvous set.

We will now present some simulation results on the approaches designed in Section 4 and Section 5. Figure 10 shows the result of both—robust optimal and stochastic optimal—signaling policies over the ‘scatter layout’ similar to the one in Figure 6. For the rescuee, the green shaded cells have a cost-to-traverse *uniformly distributed* over [2.5,3] and all other cells have a cost-to-traverse *uniformly distributed* over [1,1.5].

It can be observed that in using the stochastic optimal signaling policy (Figure 10b) the rescuer was able to exploit the distributional knowledge over the edge-weights to achieve a more optimal (in terms of cost-to-rescue) outcome. However, this comes with a penalty in terms of higher computational costs compared to the robust optimal signaling policy (Figure 10a).

In Figure 10, we assumed that the rescuee was indeed taking shortest paths. However, we saw earlier in Section 3.3 that when this assumption fails, our signaling policy from Section 2 performs very poorly. Table 2 gives the results of simulation runs showing the performance of the approach developed in Section 5.2 when the rescuee plans her path using ϵ-shortest paths (ESP), true shortest paths (SP), or vector based navigation (VBN) path planning models. We will call these the ‘True human model’. Two metrics are defined to measure performance: frequency of successful rescue and average cost of successful rescue. A rescue attempt is deemed successful if the rescue happens at the point planned for by the rescuer. The cost of rescue is computed as the sum of path costs of the rescuer and the rescuee to the rendezvous point in case of a successful rescue.

As expected, our algorithm performs very well when the rescuee is indeed taking a shortest path (SP). However, we see a sharp drop in number of successful rendezvous when the she plans her path using ESP or VBN approaches. Whether or not our approach is successful when the rescuee employs the VBN planning approach is strongly dependent on the topological layout of the rescue region. Table 3 then shows the result of the simulations runs using the approach presented in Section 5.3, i.e., when the rescuer assumes that the human takes an ESP instead of a SP.

We observe that the frequency of success is starkly higher in Table 3. Naturally, when the rescuer models the rescuee as taking ESP, the approach is more conservative as the search for candidate rendezvous points happens over larger number of paths. This conservativeness can be seen above, where the cost of rescue is higher when the rescuer considers an ESP model for the rescuee. This approach also comes with a penalty of added time complexity. Shortest paths can be computed in O(|E|+|V|log|V|) using a heap implementation Dijkstra’s algorithm. However, Yen’s algorithm we are using here has a time complexity of O(k|V|(|E|+|V|log|V|)).

## 7. Discussion and Conclusions

We began our work with a motivation to design a game theoretic framework for exploiting signaling capabilities in autonomous agents. We considered a particular SAR scenario and designed a optimal signaling policy that the rescuer can implement to achieve a desirable outcome. We then considered some scenarios where we were either missing information about the rescue environment or had incorrect assumptions about the nature of the rescuee’s behavior in Section 3. This study motivated the need for robustification of our approach and in Section 4 we developed a signaling approach that was robust to both velocity and path cost uncertainty. In doing so, we also presented a novel and efficient algorithm to obtain robust candidate rendezvous points, that is, the set of points that lie on all shortest paths irrespective of the edge-weights over the graph.

However, naturally, such a robust approach can lead to over-conservativeness in some scenarios. With the aim of exploiting additional distributional information we may have about the edge-weights over our rescue topology, we designed a stochastic optimal counterpart to the robust signaling policy in Section 5. Such a stochastic approach naturally relied on Monte Carlo methods and was computationally expensive. So the choice between using either the completely robust approach and the stochastic approach boils down to a trade-off between conservativeness and computational expense.

Finally, in a bid to better capture human behavior we looked at some additional path planning models for human agents. Supported by evidence that humans general take some ϵ-shortest path between two nodes, we designed a modification to the stochastic optimal signaling policy accounting for such path planning behavior. Once again, such a modification makes our approach more conservative, but if we are relatively certain that the rescuee is indeed not perfect in her ability to compute shortest paths, then such robustification is crucial in a success critical scenario such as ours.

In the work we presented thus far, we only considered a single signal being sent by the rescuer. Specifically, we did not attempt to re-communicate our intent in the event of a failed rescue. Although this possibility is non-existent when there is no uncertainty in edge-weights or when there is uncertainty and the rescuer implements the robust optimal signaling policy, there is always a small probability of failure when the rescuer implements the stochastic optimal signaling policy. We can then consider a multi-stage Stackelberg game, wherein the rescuer signals its intent to the rescuee multiple times. Additionally, the ability to signal multiple times in the rescue scenario might open up a richer set of strategies that the rescuer can choose from to achieve a successful rescue. Such strategies might plan for earlier messages keeping in mind the ability of the latter messages to further influence human motion. Finally, we always assumed that the human would interpret the message sent by the rescuer as intended and never considered the failure of this assumption. Although failure in rescue due to signal misinterpretation is subtly different from a failure due to uncertainty in assumed knowledge (such as the ones we addressed in our work), repeated signaling can act as a ‘corrective’ measure in both kinds of circumstances. All these avenues can be explored by considering a repeated game setting and form interesting directions for future work.

## Figures and Tables

**Table 1:** Variation of $m_{opt}$, $U_{R}$ and the rendezvous point $v_{x}$ with increasing $k_{v}$. $v_{x}$ denotes the position of a grid square using the coordinates of its bottom left corner.

$k_{v}$	$m_{\mathrm{opt}}$	$U_{R}$	$v_{x}$
1.3	R	16	(5,11)
1.6	L	15	(5,3)
1.9	R	14	(5,10)
2.5	L	10	(5,5)
3.1	R	8	(5,7)

**Table 2:** Results from simulation runs when human takes shortest paths. The results below are taken using 20 sampled runs over the topologies described in Figure 5 (strips layout) and Figure 6b (scatter layout) assuming uniform distribution of edge-weights over their supports.

Topological Layout	True Human Model	Rescuer’s Human Model	Frequency of Success	Avg. Cost of Rescue
strips	ESP	SP	8	20.46
strips	SP	SP	20	18.99
strips	VBN	SP	0	NA
scatter	ESP	SP	9	19.01
scatter	SP	SP	20	18.92
scatter	VBN	SP	20	18.92

**Table 3:** Results from simulation runs when human takes ϵ-shortest paths. The results below are taken using 20 sampled runs over two topological layout. Again we assume uniform distribution of edge-weights over their supports.

Topological Layout	True Human Model	Rescuer’s Human Model	Frequency of Success	Avg. Cost of Rescue
strips	ESP	ESP	18	21.05
strips	SP	ESP	18	21.20
strips	VBN	ESP	16	21.22
scatter	ESP	ESP	20	23.35
scatter	SP	ESP	20	22.56
scatter	VBN	ESP	20	22.57

**Figure A1:**
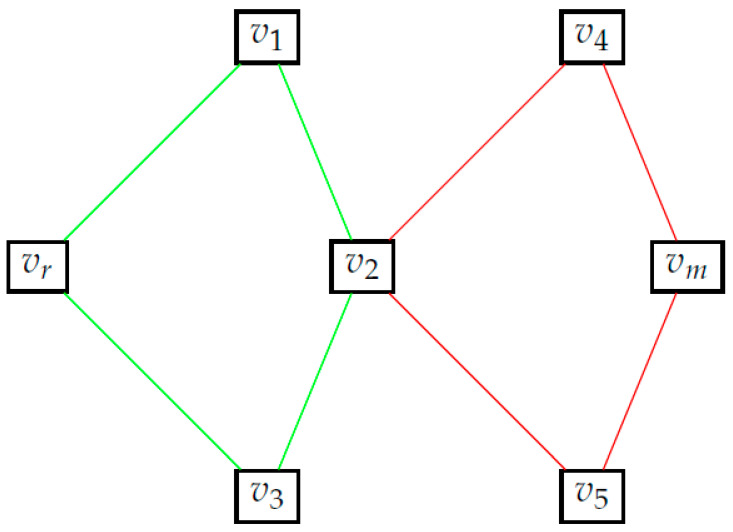
A possible representation of $G^{1}$. All edge weights are minimum. ${\hat{X}}^{1}$ would contain $\left\{v_{r},v_{2},v_{m}\right\}$. $v_{2}$ here connects the two sub-graphs red and green.

**Figure A2:**
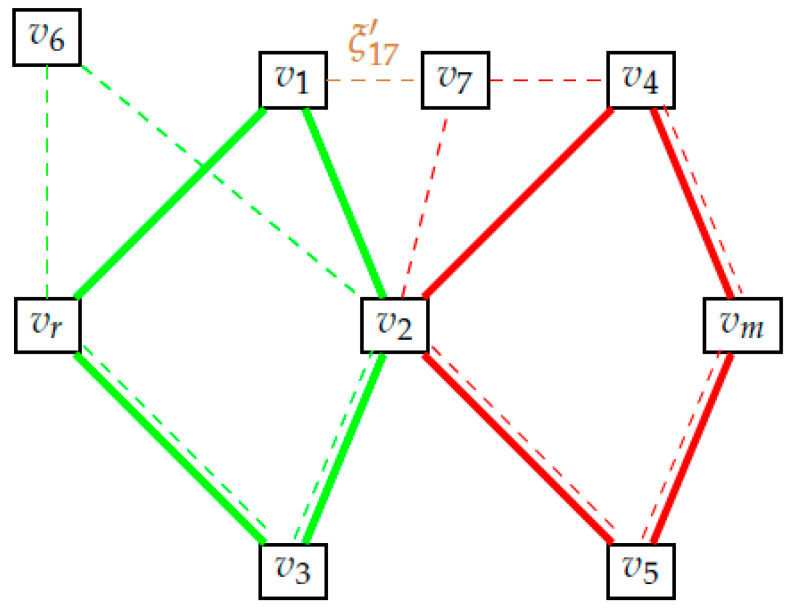
A possible representation of $G^{k}$. All thick edge weights are maximum and all thin edges are minimum weight. ${\hat{X}}^{k}$ would contain $\left\{v_{r},v_{2},v_{m}\right\}$. $v_{2}$ here connects the two sub-graphs red and green. The dashed lines indicate edges in $G^{*k}$ sub-graph. We want to show that $\xi_{17}^{\prime }$ cannot exist for any edge-weights $\left\{w_{ij}^{k^{\prime }}\right\}$.

## Data Availability

Not applicable.

## Abbreviations

The following abbreviations are used in this manuscript:

HRI	Human robot interaction
SAR	Search and rescue
UAV	Unmanned aerial vehicle
ESP	ϵ-shortest path
VBN	Vector based navigation
LOS	Line-of-sight
SP	Shortest path

## Appendix A. Proof of Correctness for the Algorithm to Find the Robust Candidate Rendezvous Points

Before we get into the proof of correctness for this algorithm it is useful to place this algorithm in a more general context. The algorithm returns the set of nodes which will always lie on shortest paths when the edge-weights are unknown but bounded. Such a problem is more ubiquitous and the algorithm has applications beyond the rescue scenario we consider. For instance, in a supply chain, when one agent is taking shortest paths over a network with travel time uncertainty and a second agent wishes to rendezvous with the first agent to exchange flow, the latter would wish to pick a point from the set of robust optimal rendezvous points. Our algorithm then computes this set efficiently in polynomial time.

It is useful to revisit some definitions of interest from graph theory before we delve into the proof.

**Definition** **A1** (Graph Union)**.** *For two graphs $G_{1}=\left(V_{1},E_{1}\right)$ and $G_{2}=\left(V_{2},E_{2}\right)$, the graph union is obtained as the new graph $G_{1}\cup G_{2}=\left(V_{1}\cup V_{2},E_{1}\cup E_{2}\right)$.*

**Definition** **A2** (Graph Compliment)**.** *Let $H=(V_{H},E_{H})$ be a sub-graph of G=(V,E), then we will define the graph compliment of H with respect to G as,*

$$G/H=(V,E/E_{H}).$$

We begin by re-introducing some of the notations used in presenting the algorithm. In doing so, we will drop the subscripts *m* and superscript *r* for increased readability. Let G=(V,E) denote our graph. Then, sub-graph $G^{*k}=\left(V^{*k},E^{*k}\right)$ denotes the acyclic digraph containing the shortest paths in the $k^{th}$ iteration of the algorithm. We can make the claim of acyclicity since all edge-weights in our graph G are assumed positive. We define a new digraph $G^{k}=\left(V^{k},E^{k}\right)$ obtained as a graph union in each iteration as,

(A1) $$G^{k}=\overset{k}{\underset{i=1}{⋃}}G^{*k}.$$

$\left\{w_{ij}^{k}\right\}$ denotes the set of edge weights at the $k^{th}$ iteration of the algorithm. Each edge-weight in $\left\{w_{ij}^{k}\right\}$ can be obtained as,

(A2) $$w_{ij}^{k}=\left\{\begin{array}{cc} {\bar{w}}_{ij} & \mathrm{if}\;\left(ij\right)\in E^{k-1}\\ {\underline{w}}_{ij} & \mathrm{if}\;\left(ij\right)\in E/E^{k-1}. \end{array}\right.$$

**Proposition** **A1.**
*Algorithm 1 return a set of nodes $\hat{X}\subseteq V$ such that every shortest path in the graph G for any set of edge-weights $\left\{w_{ij}\right\}$ passes through every node in $\hat{X}$.*

In proving Proposition A1, we first present two lemmas.

**Lemma** **A1.**
*Consider the graph $G^{k}$ and let $w_{ij}^{k^{\prime }}$ be any set of edge-weights on $G^{k}$ satisfying the property*

$$w_{ij}^{k^{\prime }}=\left\{\begin{array}{cc} w_{ij}^{\prime } & \mathrm{if}\;\left(ij\right)\in E^{k-1}\;\mathrm{and}\;{\underline{w}}_{ij}\leq w_{ij}^{\prime }\leq {\bar{w}}_{ij}\\ {\underline{w}}_{ij} & \mathrm{if}\;\left(ij\right)\in E^{k}/E^{k-1} \end{array}\right.$$

*for some set of $w_{ij}^{\prime }$s. Then, every shortest path on such a graph $G^{k}$ passes through every node in ${\hat{X}}^{k}$.*

We defer the proof of this Lemma to later.

**Lemma** **A2.**
*If $G^{*k+1}$ is a subgraph of $G^{k}$, then edges in $G/G^{k}$ are never a part of the shortest path over G. In particular, change in edge-weights over edges in $G/G^{k}$ has no effect on the shortest path in G.*

**Proof** **of Proof of Lemma A2.** Recall that $G^{*k+1}$ is obtained as the set of shortest paths when the edge-weights are $\left\{w_{ij}^{k+1}\right\}$. In this scenario, all edges in $G^{k}$ have maximum edge weight and all edges in $G/G^{k}$ have minimum edge weight. Since, the shortest path lies entirely in $G^{*k+1}$, and, thus, in $G^{k}$, any path that exits the graph $G^{k}$ is necessarily longer than the shortest path. Further, any changes in edge-weights in $G/G^{k}$ will only increase the weight of such a path. Effectively the edges in $G/G^{k}$ play no role in determining the shortest path for any value of edge-weights. Thus, all shortest paths in G are restricted to the subgraph $G^{k}$. □

**Proof** **of Proposition A1.** We defined the set $F^{k}$ as,

$$F^{k}=\left\{ij:w_{ij}^{k}={\underline{w}}_{ij},ij\in E^{*k}\right\}.$$

If the termination criteria $F^{k}=⌀$ is satisfied then all edges in $G^{*k}$ are present in $G^{k-1}$. By Proposition A2 all shortest paths lie in $G^{k-1}$ for any edge-weights over edges in $G/G^{k-1}$. Additionally, since $G^{*k}$ is a subgraph of $G^{k-1}$, we have $G^{k-1}=G^{k}$. Thus, all shortest paths lie in $G^{k}$ for any edge-weights over edges in $G/G^{k}$. By Proposition A1 we saw that all shortest paths in $G^{k}$ pass through all nodes in ${\hat{X}}^{k}$ for any value of edge weight in $G^{k-1}$. We saw above that at termination $G^{k-1}=G^{k}$, so equivalently all shortest paths in $G^{k}$ pass through all nodes in ${\hat{X}}^{k}$ for any value of edge weight in $G^{k}$. Since at termination, all shortest paths in G lie entirely in $G^{k}$ we have our result. □

Before we prove Lemma A1 we present an additional Lemma we will use in its proof. $v_{r}$ is the initial node of the rescuee and $v_{m}$ is the target node indicated by the rescuer’s signal.

**Lemma** **A3.**
*Any node $v\in {\hat{X}}^{k}$ divides the graph $G^{k}$ into two subgraphs $G_{1}^{k}$ and $G_{2}^{k}$ with $v_{r}\in V_{1}^{k}$ and $v_{m}\in V_{2}^{k}$, such that v is the only common node, i.e., $V_{1}^{k}\cap V_{2}^{k}=\left\{v\right\}$.*

**Proof** **of Lemma A3.** Let $v^{\prime }$ be another node that is common to both sub-graphs $G_{1}^{k}$ and $G_{2}^{k}$. Recall that every path in the graph $G^{*k}$ is a shortest path from $v_{r}$ to $v_{m}$ in G with edge-weights $\left\{w_{ij}^{k}\right\}$. Since $G^{k}={⋃}_{i=1}^{k}G^{*k}$, every node in $G^{k}$ must be a part of a shortest path for some set of edge-weights. Thus, we can find at least one path $\xi_{v_{r}\to v_{m}}^{\prime }$ that passes through $v^{\prime }$, such that it forms the shortest path for some set of edge-weight $\left\{w_{ij}^{l}\right\}$ for some l≤k. Since, $G^{k}$ is an acyclic digraph with all paths originating from $v_{r}$ and terminating at $v_{m}$, we cannot have any path that travels from $G_{2}^{k}$ to $G_{1}^{k}$. Thus, the path $\xi_{v_{r}\to v_{m}}^{\prime }$ containing node $v^{\prime }$ cannot also contain *v*. We showed that $\xi_{v_{r}\to v_{m}}^{\prime }$ is a shortest path on the graph G for some value of edge-weights $\left\{w_{ij}^{l}\right\}$ and that does not pass through *v*. However, such a *v* cannot lie in $\hat{X}$ by definition. Thus, by contradiction we have shown we cannot have another node $v^{\prime }$ common to both graphs $G_{1}^{k}$ and $G_{2}^{k}$. □

An example of a node in ${\hat{X}}^{k}$ dividing the $G^{k}$ into two subgraphs is shown in Figure A1. As a direct result from Lemma A3 we have,

**Corollary** **A1.**
*Any path between nodes $v_{r}$ and $v_{m}$ lying completely in graph $G^{k}$ passes through every node in ${\hat{X}}^{k}$.*

**Proof** **of Lemma A1.** From the algorithm we see that any shortest paths over the graph $G^{k}$ with edge-weights $\left\{w_{ij}^{k}\right\}$ necessarily passes through every points in ${\hat{X}}^{k}$. We wish to show the same holds true for edge-weights $\left\{w_{ij}^{k^{\prime }}\right\}$. For these edge-weights let us assume there exists a shortest path $\xi_{v_{r}\to v_{m}}^{\prime }$ that does not lie entirely in $G^{k}$. By Corollary A1, if we show that such a shortest path exiting $G^{k}$ cannot exist then we have completed the proof. Let *s* and *t* denote the node where the path $\xi^{\prime }$ leaves and rejoins the graph $G^{k}$. One such a path is illustrated in Figure A2. We know that it must leave and rejoin as both the start $\left(v_{r}\right)$ and the end $\left(v_{m}\right)$ are a part of the graph. It may leave and return to the sub-graph $G^{k}$ multiple times but for the purpose of this proof we can without loss of generality assume it does so just once each. This assumption is justified at the end of this proof. Now, let $\xi_{s\to t}^{\prime }$ denote the slice of the path that is outside $G^{k-1}$. We can find a path between *s* and *t* entirely in the graph $G^{k-1}$ as well and denote such a path as $\xi_{s\to t}^{*}$. Since, $\xi^{\prime }$ is the shortest path with edge-weights $\left\{w_{ij}^{k^{\prime }}\right\}$ we have,

(A3) $$\phi_{w^{\prime }}\left(\xi_{s\to t}^{\prime }\right)\leq \phi_{w^{\prime }}\left(\xi_{s\to t}^{*}\right).$$

where $\phi_{w^{\prime }}\left(\xi \right)$ gives the path cost of path ξ with weights $\left\{w_{ij}^{k^{\prime }}\right\}$. Now, increasing the weights in the graph $G^{k-1}$ to go from the set $\left\{w_{ij}^{k^{\prime }}\right\}$ to $\left\{w_{ij}^{k}\right\}$ will still maintain the inequality (A3), as the left hand side is not affected by the change in costs of edges in the $G^{k-1}$ and the right hand side is increasing with $\left\{w_{ij}\right\}$.

(A4) $$\Rightarrow \phi_{w^{k}}\left(\xi_{s\to t}^{\prime }\right)\leq \phi_{w^{k}}\left(\xi_{s\to t}^{*}\right)$$

where $\phi_{w^{k}}\left(\xi \right)$ gives the path cost of path ξ with weights $\left\{w_{ij}^{k}\right\}$. However, (A4) implies that there exists a shorter path outside graph $G^{k}$ (and thus outside $G^{*k}$) which is not possible. Thus, any shortest path over edge-weights $\left\{w_{ij}^{k^{\prime }}\right\}$ must lie in the graph $G^{k}$. Specifically, by Corollary A1 it must pass through all nodes $v\in {\hat{X}}^{k}$.

In closing this proof we make a comment on the assumption made on $\xi^{\prime }$ above, that it exits the graph $G^{k-1}$ at-most once. If it does exit and enter multiple times we can define $\xi_{s\to t}^{\prime }$ as a collection of splices $\left\{\xi_{s_{i}\to t_{i}}^{\prime }:i\in \left[K\right]\right\}$ where K denotes the number of splices of ξ outside $G^{k-1}$. We can consider a corresponding collection $\xi_{s\to t}^{*}=\left\{\xi_{s_{i}\to t_{i}}^{*}:i\in \left[K\right]\right\}$ of splices within the graph $G^{k-1}$ and the same proof holds with minor changes in vocabulary used. □

## Appendix B. Proof for Some Results

**Proof** **of Proposition 2.** Let $\xi_{R}^{*}\left(v_{a},v_{b}\right)={arg min}_{P\in P_{v_{a}\to v_{b}}}\phi_{R}\left(P\right)$ denote the shortest path on the graph between any two nodes $v_{a}$ and $v_{b}$ for the rescuer. Likewise $\xi_{r}^{*}\left(v_{a},v_{b}\right)$ denotes the shortest path for the rescuee over the graph between the two nodes. Then,

$$\begin{array}{cc} \frac{\phi_{R}^{*}\left(v_{x,n}\right)}{\phi_{r}^{*}\left(v_{x,n}\right)} & =\frac{\phi_{R}\left(\xi_{R}^{*}\left(v_{R},v_{x,n}\right)\right)}{\phi_{r}\left(\xi_{r}^{*}\left(v_{r},v_{x,n}\right)\right)}\\ & \leq \frac{\phi_{R}\left(\xi_{R}^{*}\left(v_{R},v_{x,m}\right)\right)+\phi_{R}\left(\xi_{R}^{*}\left(v_{x,m},v_{x,n}\right)\right)}{\phi_{r}\left(\xi_{r}^{*}\left(v_{r},v_{x,m}\right)\right)+\phi_{r}\left(\xi_{r}^{*}\left(v_{x,m},v_{x,n}\right)\right)}. \end{array}$$

Since $v_{x,m}$ also lies on the shortest path for the rescuee we have $\phi_{r}\left(\xi_{r}^{*}\left(v_{r},v_{x,n}\right)\right)=\phi_{r}\left(\xi_{r}^{*}\left(v_{r},v_{x,m}\right)\right)+\phi_{r}\left(\xi_{r}^{*}\left(v_{x,m},v_{x,n}\right)\right)$. Since $\xi_{R}^{*}\left(v_{R},v_{x,n}\right)$ is the shortest path for the rescuer to $v_{x,n}$ we have the triangle inequality $\phi_{R}\left(\xi_{R}^{*}\left(v_{R},v_{x,n}\right)\right)\leq \phi_{R}\left(\xi_{R}^{*}\left(v_{R},v_{x,m}\right)\right)+\phi_{R}\left(\xi_{R}^{*}\left(v_{x,m},v_{x,n}\right)\right)$. Hence we get

$$\begin{array}{cc} \frac{\phi_{R}^{*}\left(v_{x,n}\right)}{\phi_{r}^{*}\left(v_{x,n}\right)} & \leq \frac{\phi_{R}\left(\xi_{R}^{*}\left(v_{R},v_{x,m}\right)\right)+\phi_{R}\left(\xi_{r}^{*}\left(v_{x,m},v_{x,n}\right)\right)}{\phi_{r}\left(\xi_{r}^{*}\left(v_{r},v_{x,m}\right)\right)+\phi_{r}\left(\xi_{r}^{*}\left(v_{x,m},v_{x,n}\right)\right)}\;\left(∵\xi_{R}^{*}\;\;\mathrm{is}\;\mathrm{the}\;\mathrm{shortest}\;\mathrm{path}\right)\\ & \leq \frac{\phi_{R}\left(\xi_{R}^{*}\left(v_{R},v_{x,m}\right)\right)+w_{\max}^{R}\left(m-n\right)}{\phi_{r}\left(\xi_{r}^{*}\left(v_{r},v_{x,m}\right)\right)+w_{\min}^{r}\left(m-n\right)}\;\left(∵w_{ij}^{R}\leq w_{\max}^{R},w_{ij}^{r}\geq w_{\min}^{r}\right)\\ & \leq \frac{k_{v}\phi_{r}\left(\xi^{*}\left(v_{r},v_{x,m}\right)\right)+k_{v}w_{\min}^{r}\left(n-m\right)}{\phi_{r}\left(\xi^{*}\left(v_{r},v_{x,m}\right)\right)+w_{\min}^{r}\left(n-m\right)}\;\left(\mathrm{Assumption}\;6\right)\\ & =k_{v}. \end{array}$$

□

**Proof** **of Proposition 3.** It is easy to see that this proposition is a direct consequence of the definitions of robust counterpart to an optimization problem as provided by Ben-Tal et al. [19]. The robust counterpart to the uncertain optimization problem presented in (6) is given by,

(A5) $$\min_{m\in M}\min_{v_{x}\in X_{m}}\max_{w_{ij}^{R},w_{ij}^{R}}\;k_{1}\phi_{R}^{*}\left(v_{x}\right)+k_{2}\phi_{r}^{*}\left(v_{x}\right)$$

(A6) $$S.T.\;\;\max_{w_{ij}^{R},w_{ij}^{R}}\;\phi_{R}^{*}\left(v_{x}\right)-k_{v}\phi_{r}^{*}\left(v_{x}\right)\leq 0.$$

We can make the following observations.

$$\begin{array}{c} \phi_{R}^{*}\left(v_{x}\right)-k_{v}\phi_{r}^{*}\left(v_{x}\right)\leq \phi_{R,\max}^{*}\left(v_{x}\right)-k_{v}\phi_{r,\min}^{*}\left(v_{x}\right). \end{array}$$

The equality holds when each $w_{ij}^{r}={\underline{w}}_{ij}^{r}$ and $w_{ij}^{R}={\bar{w}}_{ij}^{r}$. Thus, if a solution satisfies (18) then it satisfies (A6) for all values of $w_{ij}^{r}$ and $w_{ij}^{r}$. In other words, such a solution is robust feasible. By similar reasoning we can see that $\max_{w_{ij}^{R},w_{ij}^{R}}\;k_{1}\phi_{R}^{*}\left(v_{x}\right)+k_{2}\phi_{r}^{*}\left(v_{x}\right)$ is attained for $w_{ij}^{r}={\bar{w}}_{ij}^{r}$ and $w_{ij}^{R}={\bar{w}}_{ij}^{r}$. Thus, the robust counterpart to (A5) subject to (A6) is given by (17) subject to (18). □

**Proof** **of Proposition 4.** We wish to show that $E_{w^{r}}\left[g\left(v,w^{r}\right)\right]=J\left(v\right)$. We will drop the $w^{r}$ arguments for shortest path taken by the rescuee $P_{m}^{*}\left(w^{r}\right)$ for the sake of brevity. By the definition of candidacy index we have,

$$\begin{array}{cc} J(v) & =E_{w^{r},P_{m}^{*}}\left[{𝟙}_{v\in P_{m}^{*}}\right]\\ & =E_{w^{r},P_{m}^{*}}\left[\sum_{P\in P_{m}^{*}\left(w^{r}\right)}{𝟙}_{v\in P}\cdot {𝟙}_{P=P_{m}^{*}}\right] \end{array}$$

where $P_{m}^{*}\left(w_{r}\right)$ denotes the set of shortest paths from $v_{r}$ to $v_{m}$ for edge-weight $w_{r}$. Then by tower property of conditional expectation we have,

$$\begin{array}{c} J\left(v\right)=E_{w^{r}}\left[\sum_{P\in P_{m}^{*}\left(w^{r}\right)}{𝟙}_{v\in P}\cdot E_{P_{m}^{*}}\left[{𝟙}_{P=P_{m}^{*}}|w_{r}\right]\right]. \end{array}$$

We made the assumption that when there are multiple shortest paths, one is chosen at random with a uniform probability over the set of all shortest paths. Given $w^{r}$, the set of shortest paths is a deterministic set. So we have,

$$\begin{array}{cc} J(v) & =E_{w^{r}}\left[\sum_{P\in P_{m}^{*}\left(w^{r}\right)}{𝟙}_{v\in P}\frac{1}{|P_{m}^{*}\left(w_{r}\right)|}\right]\\ & =E_{w^{r}}\left[g\left(v,w^{r}\right)\right]. \end{array}$$

□
